# Sugary Endosperm is Modulated by *Starch Branching Enzyme IIa* in Rice (*Oryza sativa* L.)

**DOI:** 10.1186/s12284-017-0172-3

**Published:** 2017-07-20

**Authors:** Yunjoo Lee, Min-Seon Choi, Gileung Lee, Su Jang, Mi-Ra Yoon, Backki Kim, Rihua Piao, Mi-Ok Woo, Joong Hyoun Chin, Hee-Jong Koh

**Affiliations:** 1 Department of Plant Science and Research Institute for Agriculture and Life Sciences, and Plant Genomics and Breeding Institute, Seoul National University, Seoul, 08826 South Korea; 2 Vegetable Crop Division, National Institute of Horticultural and Herbal Science, Rural Development Administration, Muan, 534-833 South Korea; 3 Department of Central Area Crop Science, National Institute of Crop Science (NICS), RDA, Suwon, 16429 South Korea; 4 Rice Research Institute, Jilin Academy of Agricultural Sciences, Gongzhuling, Jilin 136100 China; 5 Graduate School of Integrated Bioindustry, Sejong University, 209, Neungdong-ro Gwangjin-gu, Seoul, South Korea

**Keywords:** Map-based cloning, *OsBEIIa*, *OsISA1*, Rice, *Sugary-h*, Sugary endosperm

## Abstract

**Background:**

Starch biosynthesis is one of the most important pathways that determine both grain quality and yield in rice (*Oryza sativa* L.). Sugary endosperm, *sugary-1* (*sug-1*), is a mutant trait for starch biosynthesis. Rice plants carrying *sug-1* produce grains that accumulate water-soluble carbohydrates instead of starch, even after maturity. Although this trait enhances the diversity of grain quality, sugary endosperm rice has hardly been commercialized due to the severely wrinkled grains and subsequent problems in milling. This study was conducted to identify the genes responsible for the *sug-h* phenotype through a map-based cloning technology.

**Results:**

We induced a mild sugary mutant, *sugary-h* (*sug-h*) through the chemical mutagenesis on the Korean *japonica* cultivar Hwacheong. Grains of the *sug-h* mutant were translucent and amber-colored, and the endosperm appeared less wrinkled than *sug-1*, whereas the soluble sugar content was fairly high. These characteristics confer greater marketability to the *sug-h* mutant. Genetic analyses indicated that the *sug-h* mutant phenotype was controlled by a complementary interaction of two recessive genes, *Isoamylase1* (*OsISA1*), which was reported previously, and *Starch branching enzyme IIa* (*OsBEIIa*), which was newly identified in this study. Complementation tests indicated that *OsBEIIa* regulated the properties of sugary endosperm*.*

**Conclusions:**

Complementary interactions between the starch biosynthesis genes *OsISA1* and *OsBEIIa* determine the mild sugary endosperm mutant, *sugary-h*, in rice. Our finding may facilitate the breeding of sugaryendosperm rice for commercial benefit.

**Electronic supplementary material:**

The online version of this article (doi:10.1186/s12284-017-0172-3) contains supplementary material, which is available to authorized users.

## Background

Rice is the staple food for more than 3 billion people globally. The endosperm is an edible part of the rice grain, and has been one of the major targets for grain geneticists and breeders to enhance grain yield and quality. Endosperm development directly regulates grain formation at the grain filling stage. Mature rice endosperm contains starch, storage proteins, lipids, and other substances. Studies on starch have been an essential focus in rice research. Starch is the primary component that makes cereal crops economically and commercially important. Starch research is also becoming increasingly relevant for industrial and manufacturing applications.

Rice starch is composed of amylose (linear α-1,4-polyglucans) and amylopectin (α-1,6-branched polyglucans). Amylopectin has a distinct fine structure called multiple cluster structure, and accounts for approximately 65–85% of storage starch (Nakamura 2002). Starch is synthesized by four enzyme classes, with multiple subunits in each class: ADP-glucose pyrophosphorylase (AGPase); starch synthase (SS); starch branching enzyme (BE); and starch debranching enzyme (DBE). Other enzymes, such as phosphorylase (Pho) and disproportionating enzyme, are thought to be involved in starch biosynthesis. BE and DBE have important roles in determining amylopectin structure. BE forms the α-1,6-glycosidic bonds of amylopectin, whereas DBE trims improper branches generated by BE (Fujita 2014).

BE isoforms are classified into two groups, BEI (RBE1) and BEII. Cereals have two BEII isozymes, BEIIa (RBE4) and BEIIb (RBE3). These isoforms are classified according to the transferred amylopectin chain length. For example, BEII transfers shorter chains than BEI, and BEIIb transfers shorter chains than BEIIa, during extended incubations (Mizuno et al. 2001). The expression patterns of BE isoforms also differ. BEI and BEIIa transcripts have been localized in the endosperm and other tissues, whereas BEIIb is expressed only in the endosperm and reproductive tissues. In rice, BEIIa is expressed earlier than either BEIIb or BEI (Mizuno et al. 2001; Ohdan et al. 2005). Previous reports designated BEIIb-deficient mutants in maize and rice as *amylose-extender* (*ae*) mutants, in which the abundance of short amylopectin chains was reduced (Kim et al. 1998; Nishi et al. 2001). Other transgenic research about *BEIIb* gene was reported that the manipulation of BEIIb activity can generate various starch type rice, containing chalky and sugary endosperm (Tanaka et al. 2004). The RNA interference results demonstrated that reduced expression of BEIIa (SBEIIa) caused increase of amylose content in wheat endosperm (Regina et al. 2006). However, the specific functional role of BEIIa has not been elucidated in rice because the seed phenotypes of BEIIa-deficient mutants and wild-type plants are not significantly different (Fujita 2014).

DBEs directly hydrolyze α-1,6-glycosidic linkages of α-polyglucans. DBEs are classified into two types in higher plants, Isoamylase (ISA1, ISA2, and ISA3) and Pullulanase (PUL). According to Fujita (2014), Isoamylase1 (ISA1)-deficient mutants (*isa1*) were called as *sugary-1* mutants in rice (*sug-1*) and maize (*su1*). These mutants have a defect in the amylopectin cluster structure, which results in the accumulation of a polymeric water-soluble polysaccharide (WSP) termed phytoglycogen, and a reduction in the starch content (James et al. 2003). There are various *sug-1* mutant types, EM series, reported by Japanese group (Nakamura et al. 1997; Nakamura et al. 1996; Wong et al. 2003). The *sug-1* locus in rice is located on chromosome 8 (Fujita et al. 1999; Yano et al. 1984). In transgenic *sug-1* rice expressing the wheat *ISA1* gene, phytoglycogen synthesis is substantially replaced by starch biosynthesis in the endosperm (Kubo et al. 2005). This result implies that ISA1 is essential for amylopectin crystallinity and biosynthesis in both rice and wheat. In maize, double mutant defective in both ISA2 and SSIII generated water-soluble glucans in the mutant endosperm, although single mutants of either ISA2 or SSIII could synthesize normal amylopectin (Lin et al. 2012). By contrast, the contribution of PUL for amylopectin trimming was much smaller than that of ISA1, and PUL function partially overlaps with that of ISA1 (Fujita et al. 2009).

Since rice grains of *sug-1* mutants primarily contain water-soluble carbohydrates instead of starch even after maturity, they have the potential value in breeding programs for diversified qualities and commercial uses. However, they have not been used in practice due to the severely wrinkled grains and subsequent problems in milling. We developed a mild-type sugary mutant in rice, *sugary-h* (*sug-h*), which displayed an intermediate phenotype between the *sug-1* mutant and wild type. Grains of the *sug-h* mutant have better quality for subsequent processing and higher yield than *sug-1*. In addition, palatability, protein and amylose content which are crucial for breeding were increased in *sug-h* mutant (Yoon et al. 2009; *sug-2* was renamed as *sug-h* to avoid confusion with Nakagami et al. (2016)). Therefore, *sug-h* mutants could be valuable for practical applications and nutritional aspects. This study performed map-based cloning to identify the genes responsible for the *sug-h* phenotype. Our results not only identified a potential resource for utilizing the sugary endosperm mutant for commercial benefit, but also may provide a new insight into starch biosynthesis.

## Results

### Phenotypic Characterization of the *sug-h* Mutant

**Fig. 1:**
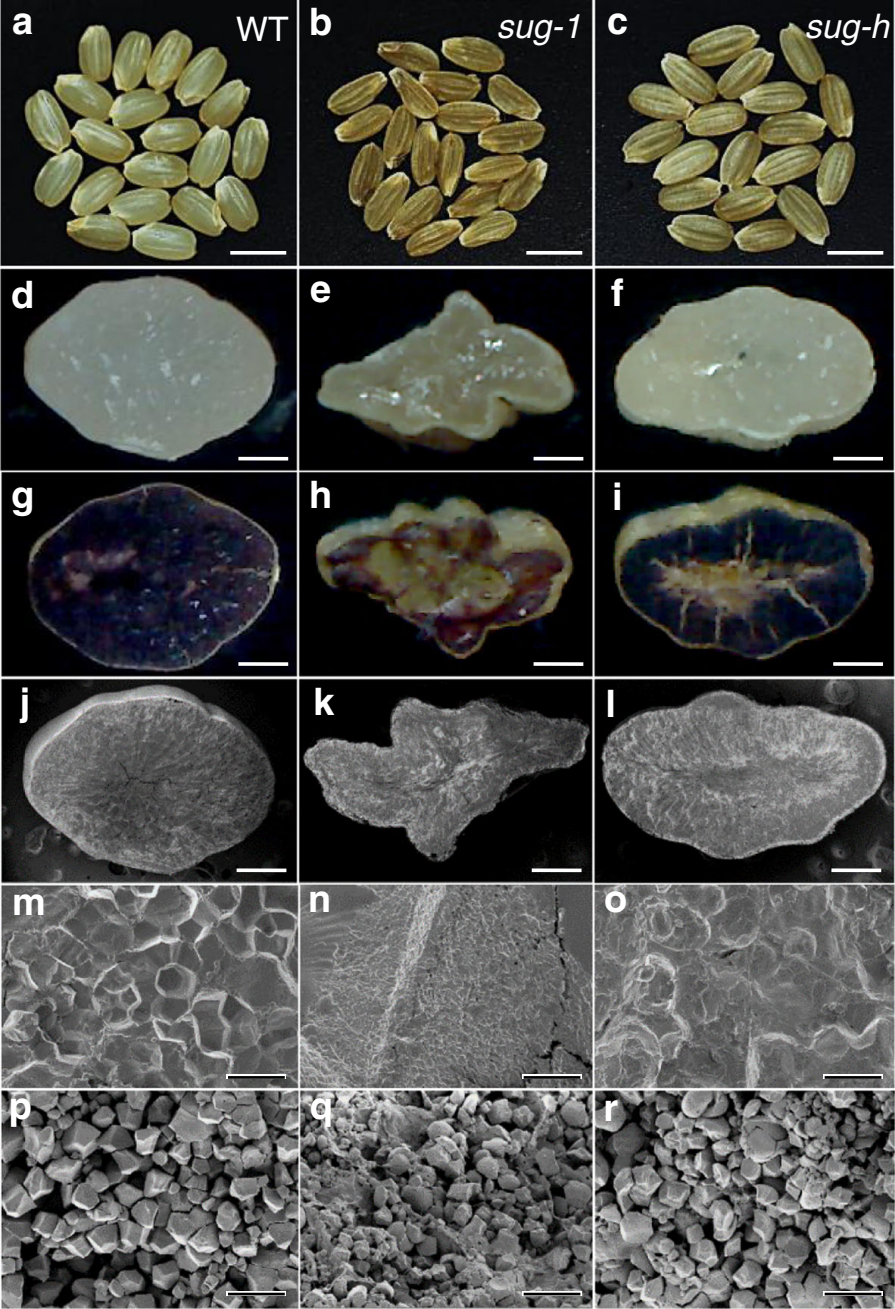
Optical and scanning electron microscopy observation of grain phenotype and starch granule structure. **a**-**c** The *sug-h* mutant grain exhibits a phenotype that is intermediate between that of the wild type and *sug-1*. Bars = 4 mm. **d**-**i** Cross sections of wild-type, *sug-1*, and *sug-h* kernels at the mature stage. Sectioned seeds were stained with iodine solution. Bars = 0.5 mm. **j**-**o** SEM observations of wild-type, *sug-1*, and *sug-h* mutant endosperm. **j**-**l** Bars = 0.5 mm. (m-o) Bars = 10 μm. **p**-**r** SEM observations of starch granule structures in wild-type, *sug-1*, and *sug-h* mutant endosperm. Bars = 10 μm

The *sug-h* mutant did not exhibit abnormal phenotypes during the vegetative stage of plant growth, although the heading date was delayed and plant height was reduced compared with that of wild-type plants (Table 1; Additional file 1: Figure S1). Grains of the *sug-h* mutant displayed an intermediate phenotype between the wild-type and *sug-1* mutant grains, that was medium thickness and slightly wrinkled (Fig. 1a-f). Wild type, *sug-1*, and *sug-h* mutants showed significant differences in grain dimensions and thickness. The grain length and width of *sug-h* mutant was slightly longer and shorter, respectively, than that of wild-type (Table 1).

**Table 1:** Agronomical characters ﻿of plant and dimensions of grain in wild type (Hwacheong) and mutants﻿

Trait	HD	CL (cm)	PN	SN	TGW (g)	GL (mm)	GW (mm)	GT (mm)	GS
WT	Aug.17	85.90 ± 1.61^a^	14.60 ± 1.43^b^	98.90 ± 16.97	19.88 ± 0.19^a^	5.11 ± 0.09^b^	3.06 ± 0.07^b^	2.17 ± 0.03^c^	1.67 ± 0.54^a^
*sug-1*	Aug.20	87.90 ± 3.04^b^	11.10 ± 3.57^a^	90.30 ± 10.86	8.76 ± 0.30^b^	4.89 ± 0.09^a^	2.96 ± 0.09^a^	1.07 ± 0.13^a^	1.65 ± 0.05^a^
*sug-h*	Aug.20	75.60 ± 1.31^c^	16.40 ± 1.84^b^	101.80 ± 9.78	14.52 ± 0.15^c^	5.41 ± 0.13^c^	2.91 ± 0.10^a^	1.70 ± 0.11^b^	1.86 ± 0.07^b^

*HD* heading date, *CL* culm length, *PN* number of panicles per plant, *SN* number of spikelets per panicle, *TGW* 1000-grain weight for brown rice, *GL* grain length, *GW* grain width, *GT* grain thickness, *GS* grain shape (length/width), *WT* wild type (Hwacheong), *sug-1 sugary-1*, *sug-h sugary-h*

Different letters denote significant differences. Ten biological replicates were used to measure for each of the traits

### Morphological Properties of Starch in Mutant Endosperm

Grains were stained with iodine to identify starch components. Phytoglycogen, which does not stain with iodine, had the highest abundance in *sug-1* endosperm. The *sug-h* mutant grain was partially stained in outermost endosperm layers, whereas the entire wild-type endosperm was stained (Fig. 1g-i). These results indicate that the endosperm starch components in both sugary mutants differed from those in the wild type, and starch production was at least partially restored in the *sug-h* endosperm.

Cross sections of polished rice grains were observed with scanning electron microscope (SEM). The images revealed that *sug-1* and *sug-h* mutants had loosely packed, abnormal starch granules in the cutting plane compared with the densely packed granules in wild-type polished grains. The *sug-h* endosperm had starch granule packing that was intermediate between that of *sug-1* and the wild type (Fig. 1j-o). Starch granules in *sug-1* and *sug-h* mutants also displayed irregular shapes with round edges, whereas those in the wild type displayed polygonal shapes with sharp edges (Fig. 1p-r). These results indicate that aberrant starch production was involved in the abnormal phenotypes of *sug-1* and *sug-h* mutants.

**Fig. 2:**
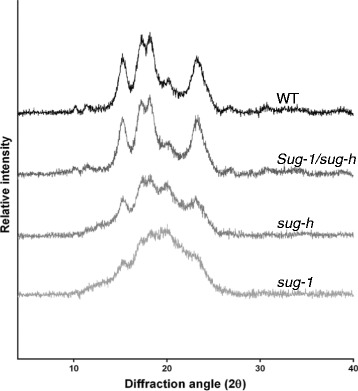
X-ray diffraction analysis of purified starch granules from mature endosperm of wild-type rice (Hwacheong), *sug-1*, *sug-h*, and *Sug-1*/*sug-h.* The seeds possessed a normal *OsISA1* and a mutated *osbe2a* genotypes were designated as *Sug-1*/*sug-h*

Starch crystallinity was investigated by performing X-ray diffraction analysis of endosperm starch from the wild type, *sug-1*, *sug-h*, and *Sug-1*/*sug-h*. Diffraction patterns of *sug-1* and *sug-h* starch displayed lower peak intensity than that of the wild type (Fig. 2). These X-ray diffraction patterns indicate that the crystallization of *sug-1* and *sug-h* starch was lower than that of wild-type starch, as reported by Yoon et al. (2009). Starch from *sug-h* mutant had an intermediate crystallization level between that from *sug-1* and the wild type. However, *Sug-1*/*sug-h*, which has a normal phenotype, displayed similar starch crystallinity to that of wild-type starch. These results indicate that *sug-1* is a major effect on the starch structure and crystallization.

### Amylopectin Fine Structure in Mature Mutant Endosperm

**Fig. 3:**
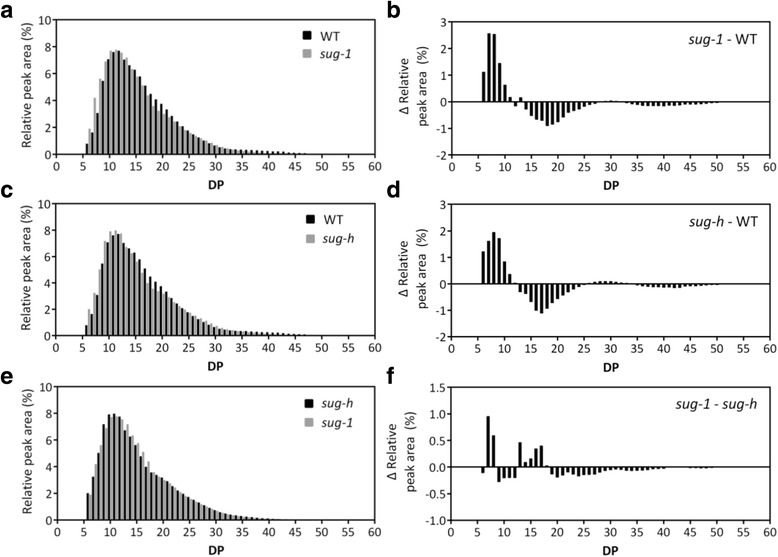
Comparison of chain length distribution of amylopectin in rice endosperm between wild type (Hwacheong) and mutant. The left panels showed chain-length profiles, and the right panels showed differences in chain-length profiles. Chain-length profiles after debranching the starch fraction and differences between WT and *sug-1* mutant (**a**-**b**), between WT and *sug-h* mutant (**c**-**d**), and between *sug-1* and *sug-h* mutant (**e**-**f**) were shown

Fine structural feature of amylopectin in wild**-**type and mutant endosperms were compared in detail by analysis of amylopectin chain-length distribution using high performance anion–exchange chromatography and a pulsed pulsed amperometric detector (HPAEC**-**PAD). Both sugary mutants had the definitely increased amount of short chains in the range of DP 6–10 (Fig. 3a–d). This result was similar to the previous reports (Nakamura et al. 1997; Wong et al. 2003; Yoon et al. 2009), indicating that mutation on ISA altered the fine structure of amylopectin into phytoglycogen in rice endosperm. The difference between *sug-1* and *sug-h* on fine structure of amylopectin was relative amount of short chains. Most range of chain-length distribution of amylopectin in *sug-h* was similar with that of *sug-1*, except in the range of DP 7–8 and DP 13–17 (Fig. 3e–f). Structural feature of amylopectin suggest that the *sug-h* phenotype was determined by the relative amount of short chains.

### Map-Based Cloning of Genes Related to the *sug-h* Phenotype

We performed map-based cloning to elucidate the genes related to the *sug-h* phenotype. Normal-type seeds were designated as ‘N-type’ and sugary-type seeds were designated as ‘S-type’. S-type seeds were grouped into ‘I-type’ (*sugary-1* type), mixed-type (I-type and II-type), and ‘II-type’ (*sugary-h* type) in segregated populations. F_2_ seeds derived from a cross between the *sug-h* mutant and Hwacheong were used for segregation ratio analysis. The results from 352 F_2_ seeds identified 271 N-type, 57 I-type, and 24 II-type seeds, which fit the expected ratio of 12:3:1 (*P* = 0.45) (Additional file 2: Table S1). Expected genotypes of N-type, I-type and II-type will be *Sug-1_ Sug-h_*, *sug-1sug-1Sug-h*_, and *sug-1sug-1sug-h sug-h*, respectively. Based on this result, we hypothesized that the *sug-h* phenotype was controlled by the epistatic interaction between two genes. One of these genes is the preceding gene, which is related to the sugary endosperm phenotype, and the other is the interacting gene, which reduces the severity of the sugary abnormality in starch biosynthesis.

**Fig. 4:**
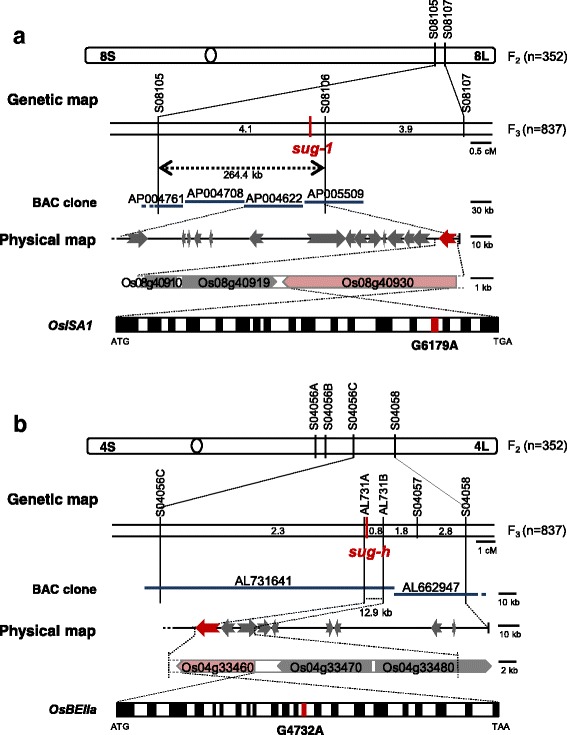
Map-based cloning of the *sug-h* mutant. **a** Candidate region of the sugary gene was on the long arm of chromosome 8. Candidate gene of the first bulked segregant analysis, Os08g40930, was located on the AP005509 BAC clone and contained 18 exons (*black boxes*) and 17 introns (*white boxes*). A point mutation, G to A, occurred in the 15th exon (*red box*). **b** Schematic representation of *OsBEIIa* on the long arm of chromosome 4. Candidate region of the gene related to the *sug-h* mutant was located within the AL731641 clone. The physical distance was approximately 12.9 kb, which included three candidate genes. Schematic structure of the candidate gene, Os04g33460, contained 22 exons (*black boxes*) and 21 introns (*white boxes*). A point mutation, G to A, occurred in the 13th exon (*red box*). *Gray arrows* indicate the main genes with known functions within the candidate region. *Red arrow* indicates the candidate gene. ATG and ﻿TGA/TAA indicate the initiation and termination codons, respectively

Preliminary mapping was initially conducted on 352 F_2_ plants derived from *sug-h*/Milyang.23 (M.23, a Korean *tongil-*type cultivar), and mapped the first gene related to the sugary phenotype. Bulked DNA for the first bulked segregant analysis (BSA) was prepared to distinguish N-type, heterozygous type (N-type and S-type), and S-type plants in the F_2_ population. Two of seventy-two sequence tagged site (STS) markers across all chromosomes, S08105 and S08107 on chromosome 8, were used as the flanking markers for fine mapping (Fig. 4a). Eighteen genes were identified in the fine-mapped region. The *OsISA1* gene sequence (Os08g40930) of Hwacheong and *sug-h* mutant was primarily compared, because previous studies reported that *OsISA1* affected the sugary phenotype in *sug-1* rice (Kubo et al. 1999; Nakamura et al. 1992; Nakamura et al. 1996). Our sequence analysis revealed that nucleotide residue 6179 in *OsISA1*, which was adenine (A) in the wild type, was substituted with guanine (G) in *sug-1* and *sug-h* mutants, thereby changing the amino acid from glycine (Gly) to aspartic acid (Asp). These results indicate that the sugary endosperm phenotype could be caused by a point mutation on *OsISA1*, which was shared by both *sug-1* and *sug-h* mutants.

One of the objectives in this study was to identify the second gene controlling the thickness and wrinkling of the sugary endosperm. Therefore, 837 F_3_ plants were derived from two individual F_2_ plants in which the *osisa1* allele was fixed, for efficient mapping. Bulked DNA for the second BSA was prepared from I-type, mixed-type (I-type and II-type), and II-type plants selected from the F_3_ population. The second BSA revealed that markers and the *sug-h* phenotype co-segregated on chromosome 4, indicating that the second gene was located on chromosome 4. For fine mapping, S04056A and S04058 were selected as flanking markers. One STS marker, S04057, and two dCAPS markers, AL731A and AL731B within the AL731641 clone, were additionally designed and applied for linkage analysis. These results reduced the size of the candidate region to 12.9 kb, which contained the following three genes: *1,4-α-glucan-branching enzyme 2*; *Ser/Thr protein phosphatase family protein*; and *histone deacetylase* (Fig. 4b). These genes were sequenced and compared in Hwacheong and *sug-h* mutant, and a point mutation was detected in the *1,4-α-glucan-branching enzyme 2* (*OsBEIIa*; *OsSBE4*) gene of the *sug-h* mutant. We found that the nucleotide residue 4732 in wild-type *OsBEIIa*, which was G, was substituted with A in the *sug-h* mutant, thereby changing the amino acid from Gly to Asp. This amino acid substitution was not found in other grain species including maize, sorghum, barley, and wheat (Additional file 3: Figure S2). These combined results indicate that the *sug-h* mutant might be caused by single point mutations resulting in amino acid substitutions in both *OsISA1* and *OsBEIIa*.

### Transgenic Complementation of the *sug-h* Mutation

**Fig. 5:**
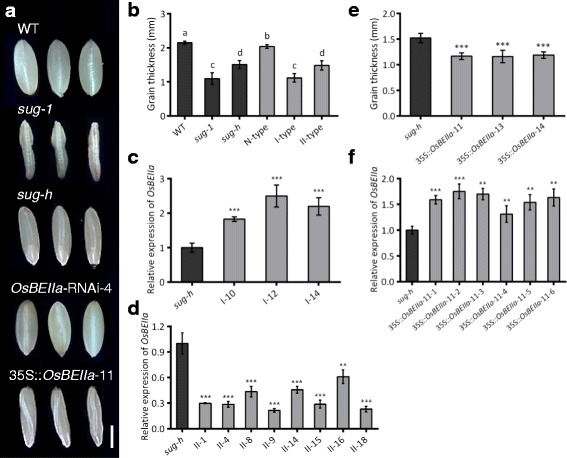
Phenotype of transgenic seeds and complementation test of the *sug-h* mutation. **a** Grain morphologies of wild-type rice (Dongjin), *sug-1*, *sug-h*, and transgenic seeds. Bar = 2 mm. **b** Comparison of grain thickness in F_2_ seeds derived from *OsBEIIa*-RNAi-4/*sug-h*. Different letters denote significant differences. Replicate samples were measured 20 times. **c** Relative expression level of *OsBEIIa* in each F_2_ plant derived from I-type seeds using qRT-PCR. The *sug-h* mutant was used as a control. Error bars represent SD for three technical experiments. Asterisks indicate statistical significance compared with the control, as determined by Student’s *t*-test (***P < 0.001). **d** Relative expression level of *OsBEIIa* in each F_2_ plant derived from II-type seeds by qRT-PCR. Error bars represent SD for three technical experiments. Asterisks indicate statistical significance compared with the control, as determined by Student’s *t*-test (**P < 0.01, ***P < 0.001). **e** Comparison of grain thickness in three lines of 35S::*OsBEIIa* and *sug-h* mutant seeds. Statistical significance was determined by Student’s *t*-test (***P < 0.001). Replicate samples were measured 20 times. **f** qRT-PCR analysis showed increased *OsBEIIa* expression in 35S::*OsBEIIa*-11 T_1_ plants. Error bars represent SD for three technical experiments. Statistical significance was determined using Student’s *t*-test (***P < 0.001)

To confirm the function of *OsBEIIa* in the *sug-h* mutant, we generated dsRNA-mediated interference (RNAi) transgenic plants. T_1_ seeds of the *OsBEIIa*-RNAi transgenic line had normal phenotype, suggesting that a single mutation of *OsBEIIa* had no effect on seed phenotype (Fig. 5a). To evaluate interactions between *OsISA1* and *OsBEIIa*, artificial crossing was performed between the *OsBEIIa*-RNAi T_0_ plant and the *sug-h* mutant. After phenotypic selection, F_2_ seeds from the artificial cross were planted for co-segregation analysis of phenotype, genotype, and RNA expression. Phenotypes of segregated F_2_ seeds showed that N-type seeds were slightly thinner than wild-type (Dongjin) seeds. By contrast, the thickness of I-type and II-type seeds was not significantly different from that of *sug-1* and *sug-h* seeds, respectively (Fig. 5b). Genotype analysis using an antibiotic resistance gene-specific primer showed that PCR bands were produced in all II-type plants, but no bands were amplified in all I-type plants (data not shown). The qRT-PCR analysis indicated that the relative *OsBEIIa* expression levels were higher in each F_2_ plant derived from I-type seed than in the *sug-h* mutant (Fig. 5c), whereas the relative *OsBEIIa* expression levels in each F_2_ plant derived from II-type seed were lower than that in the *sug-h* mutant (Fig. 5d). These results show that phenotype, genotype, and RNA expression co-segregate in the *OsBEIIa*-RNAi-4/*sug-h* F_2_ population, indicating that the two genes are associated with the *sug-h* phenotype.

We also generated transgenic plants overexpressing *OsBEIIa*, in which a vector was introduced into the *sug-h* mutant to complement the phenotype. According to the mapping results that the *sug-h* mutant might be caused by single point mutations in both *OsISA1* and *OsBEIIa* genes, this complementation test was intended to reconstruct the *sug-1* phenotype from *sug-h* mutant. Most T_1_ seeds were thinner and more wrinkled than *sug-h* seeds (Fig. 5a, e). The qRT-PCR analysis indicated that *OsBEIIa* transcript levels were higher in transgenic T_1_ plants than in *sug-h* mutant (Fig. 5f). These complementation data suggest that *osbe2a* may improve the *sug-h* phenotype by producing grains that are less wrinkled.

### Gene Expression Patterns of *OsBEIIa*

**Fig. 6:**
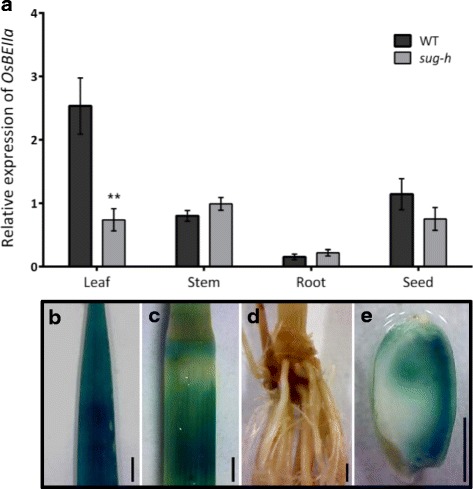
Expression pattern of *OsBEIIa* gene (**a**) qRT-PCR analysis detected *OsBEIIa* transcripts in leaf, stem, root, and 10 DAF seeds. Data are mean ± SD (*n* = 3). Statistical significance was determined using Student’s *t*-test (**P < 0.01). WT, wild-type rice (Hwacheong); DAF, days after flowering. **b**-**e** GUS expression was detected in leaf, root, stem base, and 20 DAF seeds in a transgenic plant expressing the *OsBEIIa* promoter::GUS reporter gene. Bars = 2 mm

To investigate the expression patterns of *OsISA1* and *OsBEIIa* in different organs, we performed qRT-PCR analyses and β-glucuronidase (GUS) reporter gene assay. The qRT-PCR analyses showed that *OsBEIIa* was expressed primarily in leaf, stem, and seed in wild-type and *sug-h* mutant plants (Fig. 6a). *OsBEIIa* expression in 10 days after flowering (DAF) seeds was slightly down-regulated in *sug-h* mutant compared with that in the wild type, although this change was not statistically significant. By contrast, *OsISA1* expression in *sug-h* seeds was significantly lower than that of the wild type, and *OsISA1* expression was similarly low in both wild-type and mutant leaf samples (Additional file 4: Figure S3). Transcript analysis revealed that the *OsBEIIa* mutation did not show typical RNA expression patterns in different samples, implying the possibility that other factors also determine the *sug-h* phenotype.

GUS was expressed under the control of the native *OsBEIIa* promoter in the wild-type background, and the results were consistent with the qRT-PCR data. GUS expression was detected in leaf, stem base, node, and 20 DAF seeds (Fig. 6b-e). The expression levels of *OsBEIIa* were negligible in roots as determined by both qRT-PCR and GUS reporter analyses.

### Debranching and Branching Enzyme Activities in Mutant Endosperm

**Fig. 7:**
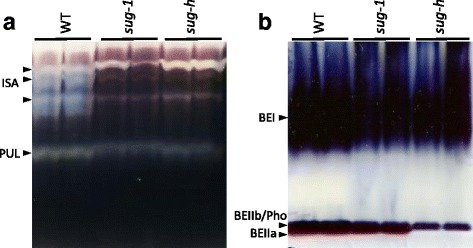
Native-PAGE/activity staining of developing endosperm in wild-type and mutant seeds. **a** Native-PAGE/debranching enzyme (DBE) activity staining of rice endosperm at late-milky stage. The ISA and PUL activity bands are indicated by black arrows. **b** Native-PAGE/branching enzyme (BE) activity staining. The BEI, BEIIa and BEIIb/Pho activity bands are indicated by *black arrows*

Native-PAGE/DBE and BE activity staining was performed to determine the change of enzyme activity in the *sug-h* mutant. Debranching enzymes, ISA and PUL, were detected as blue bands on the native gel containing potato tuber amylopectin stained with an iodine solution. ISA was visualized as three major bands with low mobility. ISA activity conspicuously decreased in both sugary mutants, as the same result with mapping on chromosome 8 (Fig. 7a).

Branching enzymes, BEI and BEII, were also detected on the native gel in distinct band patterns by iodine staining. BEI and BEIIb/Pho activities were shown in both wild-type and *sug-1* mutant. However, BEIIa activity shown as reddish bands, dramatically decreased only in the *sug-h* mutant (Fig. 7b). Zymogram results imply that the difference between *sug-1* and *sug-h* mutant was caused by branching enzyme activity.

## Discussion

Genetic mapping of the *sug-h* rice mutant was used to identify and isolate two recessive genes, *OsISA1* and *OsBEIIa*. The *sug-h* mutant has a mild sugary phenotype, which preferentially accumulates desirable sugar compositions of non-starch polysaccharide rather than starch, and is more commercially viable than the *sug-1* mutant because it does not display excessive wrinkling, which interferes with milling (Koh and Heu 1994; Yoon et al. 2009). Segregation ratios of the F_2_ population showed that the *sug-h* phenotype was controlled by complementary interactions between *OsISA1* and *OsBEIIa*. We demonstrated that *OsISA1* and *OsBEIIa* were associated with the genetic modifications that were responsible for the sugary endosperm phenotype. Although a single mutation in *OsBEIIa* did not affect endosperm phenotype, the mutation in *OsBEIIa* moderately recovered the sugary endosperm from the severe wrinkling caused by *osisa1*. Therefore, *sug-h* mutant seed maintains a sugary phenotype, but the seed quality is superior (less wrinkled) than that of *sug-1*.

There have been several reported mutants and transgenic rice related to sugary-type endosperm. Among them, severe sugary-type endosperm mutants, such as EM914 (Nakamura et al. 1997; Wong et al. 2003), #1–1 (Tanaka et al. 2004), and *OsISA1* suppression and *OsISA2* over-expression transgenic lines (Utsumi et al. 2011), were similar to the *sug-1* mutant used in this study. Of them, phenotype of EM914 was governed by mutated *ISA1* while #1–1 and *OsISA2* over-expression line had different genes than *sug-1*. It is interesting that a mild sugary mutant, which was reported as a variation of *su-1* mutant by Nakamura et al. (1997), had the similar phenotype to *sug-h* mutant although only *sug-1* locus was involved in the *su-1* mutant. The reason for the phenotypic similarity between *su-1* mutant by Nakamura et al. (1997) and *sug-h* mutant in this study remains to be comparatively studied. Recently, a rice novel endosperm mutant, named as *sugary-2*, was reported (Nakagami et al. 2016), in which the results on the activity of BE in the *sugary-2* mutant was unlike the *sug-h* mutant, indicating that the *sugary-2* mutant was different from the *sug-h* mutant.

Nakamura (2002) reported that inhibition of BEIIa activity caused low levels of short amylopectin chains with degree of polymerization (DP) ≤10 in rice leaf sheath, in which BEIIb is not expressed; however, the BEIIa-deficient mutant does not exhibit a significant change in amylopectin chain length profile in rice endosperm. Because a direct function of the *OsBEIIa* could not have been elucidated yet, we suggest that the amino acid substitutions in *OsISA1* and *OsBEIIa* changed the protein complex or enzyme interaction involved in starch biosynthesis, and might be responsible for the *sug-h* phenotype affecting amylopectin structure. Future studies will perform enzymatic analyses to test this hypothesis.

Previous studies investigated possible interactions between ISA and other enzymes. The debranching enzyme PUL was related to the sugary phenotype (Fujita et al. 2009). Amylose content, seed morphology, and starch granules of *pul* mutant lines were essentially the same as those of wild-type plants. By contrast, double *pul* and *isa1* mutant lines contained higher levels of WSPs and had shorter amylopectin chains with DP ≤7 in the endosperm compared with the *sug-1* parents, indicating that PUL can partly compensate for starch biosynthesis. The absence of ISA activity primarily affected the sugary endosperm phenotype regardless of the presence of PUL activity. This result was very similar using the *sug-h* mutant; however, no differences in *OsPUL* sequences were identified in the Hwacheong wild type and the *sug-h* mutant.

*FLOURY ENDOSPERM6* (*FLO6*) encodes a CBM48 domain-containing protein (Peng et al. 2014). FLO6 may act as a starch-binding protein interacting with ISA1, and may be a bridge between ISA1 and starch during starch biosynthesis. ISA1 may have interacting factors that mediate starch binding, and interacting enzymes that have not yet been elucidated. Previous research evaluated protein-protein interactions of starch biosynthetic enzymes. Crofts et al. (2015) reviewed that co-immunoprecipitation analysis revealed the following associations in rice: BEIIa-BEIIb, BEIIa-BEI, BEIIa-Pho1, BEIIa-SSI, and BEIIa-SSIIIa. The BEIIa-SSI interaction was also identified in wheat and maize (Tetlow et al. 2008). These results suggested that some isozymes involved in starch biosynthesis in rice formed active protein complexes. These combined results suggest a possible mechanism of BEIIa function in rice endosperm.

Phenotypic variation is a critical consideration for phenotypic analysis of sugary endosperm in *sug-h* populations because of environmental effects. Satoh et al. (2008) evaluated the effect of growth temperature on the frequency of various grain phenotypes and the extent of starch accumulation in the wild type and mutant, and reported that starch accumulation in the *phosphorylase1* (*pho1*) mutant endosperm was affected by temperature. Similarly, the seed phenotypes of *sug-1* and *sug-h* mutants differed slightly between plants grown in the field and those grown in the green house (data not shown). To reduce this phenotypic variation, all seeds from a whole main panicle of F_2_ and F_3_ plants grown in the field were used for genetic mapping. The mutants and wild-type seeds were grown together under the same conditions and prepared for phenotypic analysis at the same time. Future studies should assess the effects of environmental factors, especially temperature, on phenotypic variation.

In this study, we propose that mutated *OsBEIIa* plays a role in restoring the severely wrinkled sugary phenotype caused by *osisa1* in rice endosperm, although *OsBEIIa* mutation alone did not result in a significant phenotypic change. The observed complementary interaction between *OsISA1* and *OsBEIIa* provides novel insight into the roles of starch biosynthesis enzymes and their interactions. Our result can facilitate the breeding of functional rice cultivars with special nutritional qualities, and might be applicable to endosperm modification in other cereal crops.

## Conclusions

Grains of *sug-h* mutant have less wrinkled feature, which make it more commercially feasible by easy polishing in processing. Here, we report the cloning of a new sugary gene in rice, which controls the thickness of sugary endosperm grains. Genetic analysis revealed that phenotype of *sug-h* mutant was controlled by a complementary interaction of two recessive genes, *OsBEIIa* and *OsISA1*. Complementation test demonstrated that sugary endosperm was modulated by *OsBEIIa*. These results may facilitate the breeding of sugary endosperm rice for commercial use and will be helpful to enlarge our understanding on starch biosynthesis in rice endosperm.

## Methods

### Plant Materials

The *sug-h* mutant was induced by N-methyl-N-nitrosourea (MNU) treatment on the Korean *japonica* cultivar Hwacheong (Koh and Heu 1994; The *sug-2* was renamed in this study as *sug-h* to avoid confusion with Nakagami et al. (2016)). The F_2_ population was derived from a cross between the *sug-h* mutant and M.23. F_3_ seeds were classified into three groups: normal, sugary, and heterozygous type. Two F_2_ individuals that displayed the sugary-type in F_3_ seeds were developed for the F_3_ population. To calculate the segregation ratio, another F_2_ population derived from a cross between the *sug-h* mutant and wild-type Hwacheong was used. The *sug-1* mutant line was selected from the *sug-h*/Hwacheong F_2_ population, and isolated to the F_6_ generation via self-pollination. Normal-type seeds were designated as ‘N-type’ and sugary-type seeds were designated as ‘S-type’. S-type seeds were grouped into ‘I-type’ (*sugary-1* type), ‘II-type’ (*sugary-h* type) and mixed-type (I-type and II-type) in segregating populations. These populations were cultivated using conventional methods at the Experimental Farm of Seoul National University.

### Phenotypic Analysis

All F_1_, F_2_, and F_3_ seeds were dehulled and observed under a microscope. S-type and N-type seeds were distinguished by seed thickness and severity of wrinkling. All seeds from a whole panicle were used for phenotyping to minimize phenotypic error resulting from differences in seed maturity. Grain dimensions including length, width, and thickness were measured using digimatic calipers (Mitutoyo, Japan) and analyzed using the IBM SPSS statistics program. Each seed was stained with iodine solution [0.1% (*w*/*v*) I_2_ and 1% (*w*/*v*) KI] to detect starch.

### Scanning Electron Microscopy

Seeds and starch samples were visualized with a SEM according to the previously published method of Fujita et al. (2003). Gold-coated seeds and starch samples were observed using a SUPRA 55VP Scanning Electron Microscope (Carl Zeiss, Germany).

### X-ray Diffraction Analysis of Starch

One of the samples for morphological analysis of starch properties, *Sug-1*/*sug-h*, possessed normal *OsISA1* and mutated *osbe2a* alleles. X-ray diffraction was used to determine the structures of starch according to the methods described previously by Kubo et al. (2005). The X-ray diffraction patterns of isolated insoluble glucans were obtained with a copper (nickel foil-filtered) K_α_ radiation using D8 Advanced X-ray diffractometry (Bruker, Germany) at 40 kv and 40 mA. The scanning region of the two-theta angle (2θ) ranged from 4.0 to 40.0° with a scan speed of 0.5 deg. min^−1^.

### Chain–Length Distribution of Amylopectin by HPAEC-PAD

The chain–length distributions of amylopectin from wild-type and mutants endosperm were analyzed using HPAEC**-**PAD as in previous reports (Hanashiro et al. 1996; Kwak et al. 2017).

### DNA Extraction and PCR Amplification

Total genomic DNA was extracted from young leaves of F_2_, F_3_ plants and their parents according to the method of McCouch et al. (1988) with modifications. Polymerase chain reactions (PCR) were performed in a reaction volume of 20 μl containing 100 ng of template DNA, 0.1 μM each primer, 2.5 mM dNTP, 10 mM Tris-HCl (pH 8.3), 50 mM KCl, 1.5 mM MgCl_2_, 0.01% (*w*/*v*) gelatin, and 0.5 U Taq DNA polymerase. PCR amplification was carried out in a DNA Engine Tetrad 2 and Dyad Thermal Cycler (Bio-Rad, USA) using the following reaction conditions: 5 min at 94 °C; followed by 35 cycles of 1 min at 94 °C, 30 s at 56 °C, and 40 s at 72 °C; and 10 min at 72 °C for final extension. PCR products were separated on 2.5% (*w*/*v*) agarose gels containing 0.15 μg ml^−1^ ethidium bromide (EtBr) in 0.5 × TBE buffer.

### Linkage Mapping of the *sug-h* Mutant

We performed BSA to genetically map and isolate genes related to the *sug-h* mutant (Michelmore et al. 1991). Ten N-type plants, ten heterozygous type plants (N-type and S-type), and ten S-type plants were selected from the *sug-h*/M.23 F_2_ population for the first BSA to identify gene distinguishing N-type and S-type. For the second BSA, ten I-type plants, 12 mixed-type plants (I-type and II-type), and 12 II-type plants were selected from the *sug-h*/M.23 F_3_ population, in which the sugary allele was fixed. Three bulked samples containing randomly combined equal amounts of DNA were used for genotyping. Then, fine mapping was conducted on two chromosomes with flanking STS markers, which were developed by designing primers based on the DNA sequence differences between *indica* and *japonica* rice cultivars (Chin et al. 2007). Additional STS and derived cleaved amplified polymorphic sequence (dCAPS) primers were designed with Primer3 version 0.4.0 (http://frodo.wi.mit.edu/primer3) for additional fine mapping based on the available rice genome sequence data (http://www.gramene.org, http://www.ncbi.nlm.nih.gov). Primer sequences and other information for DNA markers designed and used in this study are listed in Additional file 5: Table S2.

### Candidate Gene Analysis

To validate the candidate gene models, full-length genomic DNA of each candidate gene in Hwacheong and the *sug-h* mutant was amplified by PCR. PCR products were purified using the PCR purification kit (Inclone, Korea) and transformed into *E. coli* strain DH5α, followed by ligation of PCR amplicons into the pGEM-T Easy Vector (Promega, USA). Transformed plasmid sequences were analyzed with an ABI Prism 3730 XL DNA Analyzer (PE Applied Biosystems, USA). The PCR clones were verified by sequence alignment with the original parent using CodonCode Aligner software (CodonCode Corporation, USA). Based on the results of the sequencing analysis, multiple alignments were performed using a public database (http://www.ch.embnet.org/software/BOX_form.html).

### RNA Isolation and Quantitative Real-Time PCR Analysis

Total RNA was extracted from various tissues of wild-type, mutant, and transgenic plants using MG Total RNA Extraction kit (Doctor Protein, Korea). The RNAs were converted into first-strand cDNA using M-MLV Reverse Transcriptase (Promega, USA). Quantitative RT-PCR was performed using SYBR Premix ExTaq (Takara Bio, Japan) according to the manufacturer’s instructions. Gene expression levels were evaluated in leaf, stem, and root samples collected at maximum tillering stage and seed samples collected at 5, 10, and 20 DAF. The following gene-specific primer sets were used: *OsISA1*-RT, 5′-CAAATGCGCAATTTCTTTGTT-3′ (sense) and 5′-GTCCCAGCGGAAATAATTGA-3′ (antisense); *OsBEIIa*-RT, 5′-GCCAATGCCAGGAAGATGA-3′ (sense) and 5′-GCGCAACATAGGATGGGTTT-3′ (antisense) (Zhang et al. 2012); control *UBQ*-qPCR, 5′-GAGCCTCTGTTCGTCAAGTA-3′ and 5′-ACTCGATGGTCCATTAAACC-3′ (Tanaka et al. 2011). Quantitative RT-PCR was performed using a C1000 thermal cycler (Bio-Rad, USA).

### Complementation of the *sug-h* Mutant

The RNA interference (RNAi) vector was constructed by PCR amplification of a 291 bp fragment from *OsISA1* and a 206 bp fragment from *OsBEIIa* cloned from Hwacheong cDNA. These fragments were subcloned into pDONR201 (Invitrogen, USA), and then transferred into the RNAi vector pH7GWIWG (II) using Gateway BP and LR clonase enzyme mixes (Invitrogen, USA). The full-length *OsBEIIa* cDNA was amplified from Hwacheong cDNA and used for constructing the overexpression vector. The amplified fragment was transferred into pMDC32 via pCR™ 8/GW/TOPO® TA Cloning Kit (Invitrogen, USA). The RNAi constructs were transformed into wild-type Dongjin (a *japonica* cultivar), and the overexpression construct was transformed into callus of the *sug-h* mutant. Transformation was performed using a modification of the previously published *Agrobacterium*-mediated transformation method (Nishimura et al. 2006).

### Histological GUS Assay

The 1909 bp region upstream from the start codon of *OsBEIIa* was amplified and cloned into the binary vector pHGWFS7 using Gateway BP and LR clonase enzyme mixes (Invitrogen, USA). The final construct was introduced into wild-type Dongjin by *Agrobacterium*-mediated transformation. Transgenic plants containing the *OsBEIIa* promoter::GUS reporter construct were selected, and T_0_ plants were used for GUS assays. GUS staining was performed as described previously (Jefferson et al. 1987). X-Gluc buffer solution was vacuum-infiltrated into several different tissue samples. The samples were incubated overnight in X-Gluc buffer solution at 37 °C, and then washed with a graded ethanol series.

### Native–PAGE/Activity Staining of DBE and BE

Crude enzyme was extracted using the method described by Yamanouchi and Nakamura (1992) from ten seeds at late**-**milky stage. Native–PAGE/activity staining of DBE was performed using the modified method of Nakamura et al. (1997). Native slab gel was prepared with 6.5% (*w*/*v*; resolving gel) containing 0.3% (*w*/*v*) potato tuber amylopectin (Sigma, USA), and 3.3% (*w*/*v*; stacking gel) acrylamide. Electrophoresis was carried out at 4 °C at constant current of 20 mA for 2.5 h. For detection of the isoamylase activity, the gel was rinsed with 20 ml of 50 mM Na-acetate (pH 5.4), 50 mM 2-mercaptoethanol and 50 mM CaCl_2_ at room temperature and then incubated at 37 °C for 3 h with 20 ml of the same buffer solution. Native–PAGE/activity staining of BE was carried out according to the modified method by Yamanouchi and Nakamura (1992). A slab gel prepared with 5% (*w*/*v*; resolving gel) and 3.3% (*w*/*v*; stacking gel) acrylamide. Electrophoresis was carried out at 4 °C at constant current of 20 mA. After electrophoresis, the gel was imbibed with 20 mL of a solution containing 50 mM HEPES-NaOH buffer (pH 7.4) and 20% (*v*/v) glycerol for 15 min on ice. For detection of BE, the gel was incubated in 20 ml of the reaction mixture, which consisted of 50 mM HEPES-NaOH buffer (pH 7.4), 50 mM Glc-1-P (Sigma, USA), 2.5 mM AMP (Sigma, USA), 10% (*v*/v) glycerol, and rabbit muscle phosphorylase a (about 30 units; Sigma, USA) for 6 h at 30 °C with gentle shaking. Iodine solution [0.1% (*w*/*v*) I_2_ and 1% (*w*/*v*) KI] was used for staining both gels.

## Additional files

Additional file 1: Figure S1. Plant and panicle morphology of wild-type rice (Hwacheong) and mutants. (a-b) Plant phenotype of wild-type and mutant plants 54 days after transplanting (a) and at the milky stage (b). (c) Panicle length of wild-type and mutant plants at the yellow ripe stage. (PDF 439 kb)

Additional file 2: Table S1. Genetic analysis of the *sug-h* mutant using F_3_ seeds (DOCX 14 kb)

Additional file 3: Figure S2. Multiple alignments of cereal OsBEIIa proteins. Protein of the *sug-h* mutant (top line) was aligned with that of wild-type rice (Hwacheong) and four cereal plants (barley, maize, sorghum, and wheat). Black boxes indicate identical residues; gray boxes indicate similar residues. Mutated region is marked with an asterisk. Color bars indicate the domains; E_set_GBE_euk_N (green), AmyAc_bac_euk_BE (blue), and Alpha-amylase_C (orange). (PDF 1064 kb)

Additional file 4: Figure S3. *OsISA1* expression patterns in different organs and at different stages of seed development using qRT-PCR analysis. (a) Transcript levels decreased in seed (10 DAF) of the *sug-h* mutant. (b) *OsISA1* expression in 10 and 20 DAF seeds decreased in the *sug-h* mutant. All data are mean ± SD (*n* = 3). Statistical significance was determined using Student’s *t*-test (*P < 0.05, **P < 0.01). WT, wild-type rice (Hwacheong); DAF, days after flowering. (PDF 51 kb)

Additional file 5: Table S2. Molecular markers used for fine mapping of the *sug-h* mutant. (DOCX 14 kb)

## Abbreviations

BEIIa: Branching enzyme IIa
BSA: Bulked segregant analysis
dCAPS: Derived cleaved amplified polymorphic sequence
ISA1: Isoamylase1
PCR: Polymerase chain reaction
STS: Sequence tagged site
sug-1: sugary-1
sug-h: sugary-h
WSP: Water-soluble polysaccharide
