# Supplementary figures and images for: Sugary Endosperm is Modulated by Starch Branching Enzyme IIa in Rice (Oryza sativa L.)
Source: Rice (N Y). 2017 Jul 20;10:33. doi: 10.1186/s12284-017-0172-3 (PMC5519516; doi:10.1186/s12284-017-0172-3)

**a**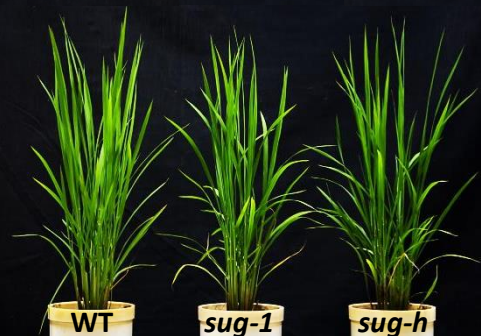**b**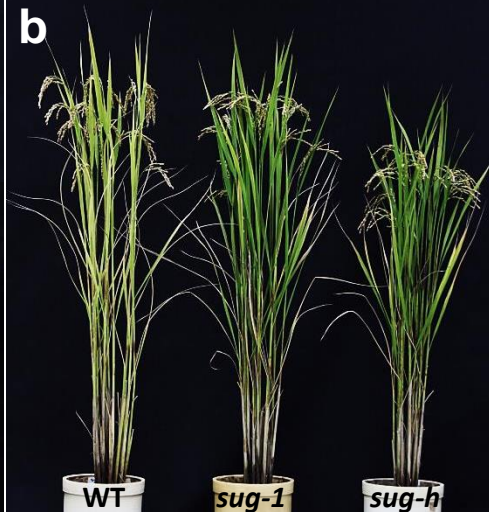**c**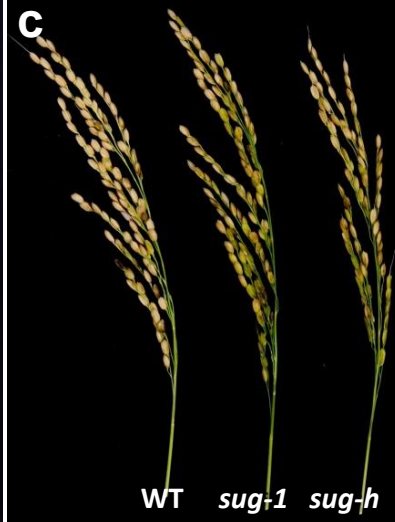

Supplement: Supplementary file 1 — Plant and panicle morphology of wild-type rice (Hwacheong) and mutants. (a-b) Plant phenotype of wild-type and mutant plants 54 days after transplanting (a) and at the milky stage (b). (c) Panicle length of wild-type and mutant plants at the yellow ripe stage. (PDF 439 kb) [file 12284_2017_172_MOESM1_ESM.pdf]

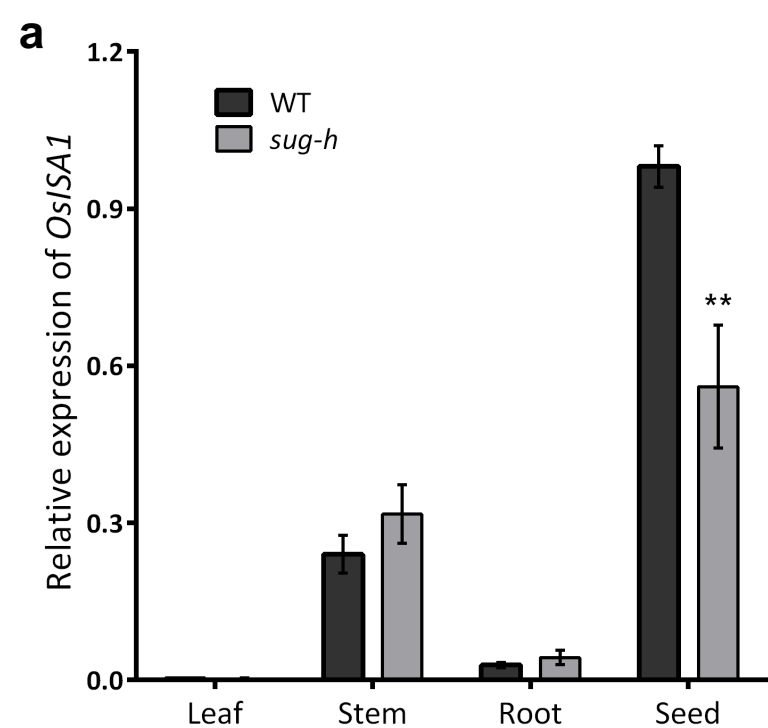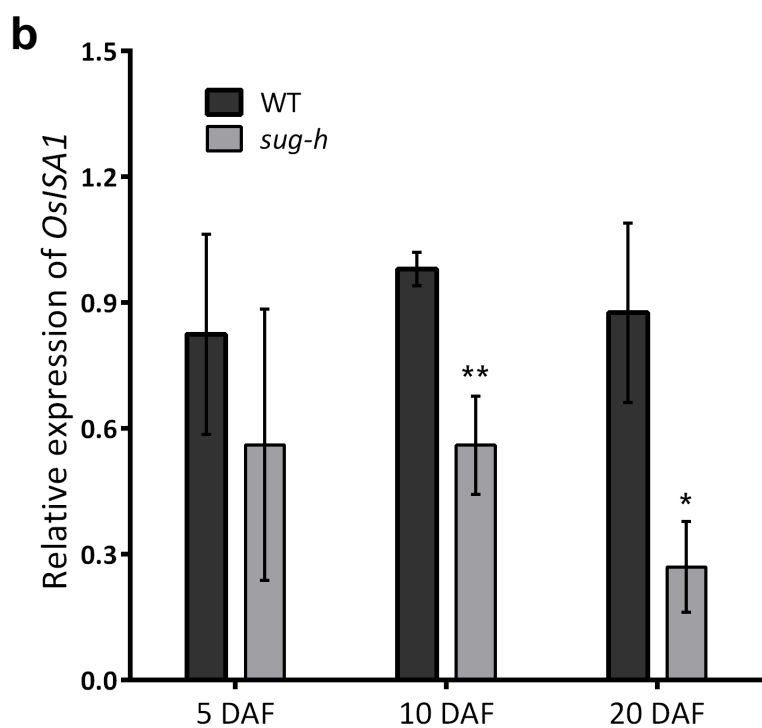

Supplement: Supplementary file 4 — OsISA1 expression patterns in different organs and at different stages of seed development using qRT-PCR analysis. (a) Transcript levels decreased in seed (10 DAF) of the sug-h mutant. (b) OsISA1 expression in 10 and 20 DAF seeds decreased in the sug-h mutant. All data are mean ± SD (n = 3). Statistical significance was determined using Student’s t-test (*P < 0.05, **P < 0.01). WT, wild-type rice (Hwacheong); DAF, days after flowering. (PDF 51 kb) [file 12284_2017_172_MOESM4_ESM.pdf]
