# Supplementary material for: Sugary Endosperm is Modulated by Starch Branching Enzyme IIa in Rice (Oryza sativa L.)
Source: Rice (N Y). 2017 Jul 20;10:33. doi: 10.1186/s12284-017-0172-3 (PMC5519516; doi:10.1186/s12284-017-0172-3)
Supplement: Supplementary file 2 — Genetic analysis of the sug-h mutant using F3 seeds (DOCX 14 kb) [file 12284_2017_172_MOESM2_ESM.docx]

**Table S1**

| **Cross combination** | **No. of seeds** | | | | $\boldsymbol{\chi}^{\boldsymbol{2}}$**_0.05_***  **(12:3:1)** | ***P* value** |
| --- | --- | --- | --- | --- | --- | --- |
|  | **N-type** | **S-type** | | **Total** |  |  |
|  |  | **I** | **II** |  |  |  |
| Hwacheong/*sug-h* | 271 | 57 | 24 | 352 | 1.595 | 0.451 |
| *sug-h*/Hwacheong | 152 | 28 | 11 | 191 | 2.312 | 0.315 |
