# Supplementary material for: Sugary Endosperm is Modulated by Starch Branching Enzyme IIa in Rice (Oryza sativa L.)
Source: Rice (N Y). 2017 Jul 20;10:33. doi: 10.1186/s12284-017-0172-3 (PMC5519516; doi:10.1186/s12284-017-0172-3)
Supplement: Supplementary file 3 — Multiple alignments of cereal OsBEIIa proteins. Protein of the sug-h mutant (top line) was aligned with that of wild-type rice (Hwacheong) and four cereal plants (barley, maize, sorghum, and wheat). Black boxes indicate identical residues; gray boxes indicate similar residues. Mutated region is marked with an asterisk. Color bars indicate the domains; E_set_GBE_euk_N (green), AmyAc_bac_euk_BE (blue), and Alpha-amylase_C (orange). (PDF 1064 kb) [file 12284_2017_172_MOESM3_ESM.pdf]

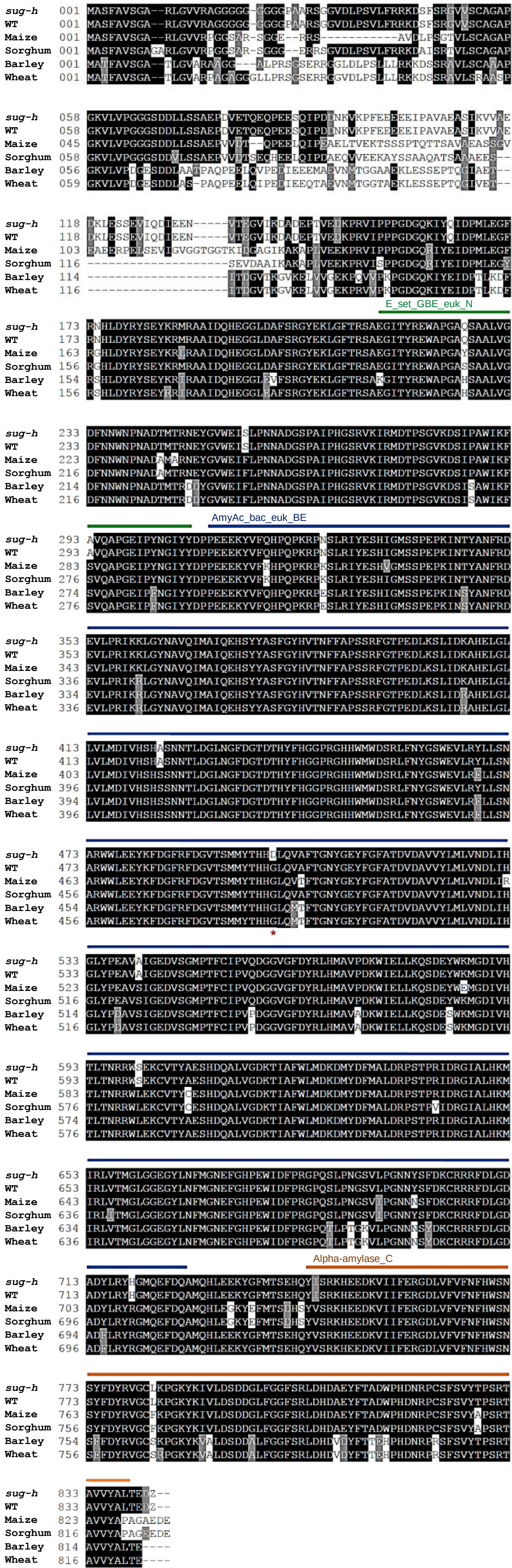

sug-h 001 MASFAVSGA--RLGVVRAGGGGG-GGGGPAARSGGVDLPSVLFRRKDSFSRGVVSCAGAP
WT 001 MASFAVSGA--RLGVVRAGGGGG-GGGGPAARSGGVDLPSVLFRRKDSFSRGVVSCAGAP
Maize 001 MASFAVSGA--RLGVVRPGGSAR-SGGE--RRS-----------AVDLPSGTVLSCAGAP
Sorghum 001 MASFAVSGAGARLGVVRPGGSAR-SGGG--DRRSGVDLPSVLFRRKDAISRTVLSCAGAP
Barley 001 MATFAVSGA--TLGVARAAGG---ALPRSGSERRGGLDLPSLLLRKKDSSRAVLSCAAAP
Wheat 001 MATFAVSGA--TLGVARPAGAGGGLLPRSGSERRGGVDLPSLLLRKKDSSRAVLSRAASP
sug-h 058 GKVLVPGGGSDDLLSSAEPDVETQEQPEESQIPDDNKVKPFEEEEIPAVAEASIKVVAE
WT 058 GKVLVPGGGSDDLLSSAEPDVETQEQPEESQIPDDNKVKPFEEEEIPAVAEASIKVVAE
Maize 045 GKVLVPGGGSDDLLSSAEPVVDT--QPEELQIPEAELTVEKTSSSPTQTTSAVAEASGV
Sorghum 058 GKVLVPGGGSDDVLSSAEPVVDTSEQHEELQIPDAEQVVEEKAYSSAAQATSAAAEES--
Barley 056 GKVLVPDGESDDLAATPAQPEELQVPEDIEEEMAEVNMTGGAAEKLESSEPTQGIAET--
Wheat 059 GKVLVPDGESDDLAS-PAQPEELQIPEDIEEQTAEVNMTGGTAEKLESSEPTQGIVET--
sug-h 118 DKLESSEVIQDIEEN-----VTEGVIKDADEPTVEDKPRVIPPPGDGQKIYQIDPMLEGF
WT 118 DKLESSEVIQDIEEN-----VTEGVIKDADEPTVEDKPRVIPPPGDGQKIYQIDPMLEGF
Maize 103 EAEERPELSEVIGVGGTGGTKIDGAGIKAKAPIVEEKPRVIPPPGDGQRIYEIDPMLEGF
Sorghum 116 --------------------SEVDAAIKAKAPIVEEKPRVISPPGDGQRIYEIDPMLEGY
Barley 114 --------------------ITDGVIKGVKEIVVGEKPQVVPKPGDGQKIYEIDPTLKDF
Wheat 116 --------------------ITDGVIKGVKEIVVGEKPRVVPKPGDGQKIYEIDPTLKDF
E_set_GBE_euk_N
sug-h 173 RNHLDYRYSEYKRMRAAIDQHEGGLDAFSRGYEKLGFTRSAEGITYREWAPGAQSAALVG
WT 173 RNHLDYRYSEYKRMRAAIDQHEGGLDAFSRGYEKLGFTRSAEGITYREWAPGAQSAALVG
Maize 163 RGHLDYRYSEYKRLRAAIDQHEGGLDAFSRGYEKLGFTRSAEGITYREWAPGAYSAALVG
Sorghum 156 RGHLDYRYSEYKRMRAAIDQHEGGLDAFSRGYEKLGFTRSAEGITYREWAPGASSAALVG
Barley 154 RSHLDYRYSEYKRLRAAIDQHEGGLEVFSRGYEKLGFTRSAKGITYREWAPGAHSAALVG
Wheat 156 RSHLDYRYSEYRRIRAAIDQHEGGLEAFSRGYEKLGFTRSAEGITYREWAPGAHSAALVG
sug-h 233 DFNNWNPNADTMTRNEYGVWEISLPNNADGSPAIPHGSRVKIRMDTPSGVKDSIPAWIKF
WT 233 DFNNWNPNADTMTRNEYGVWEISLPNNADGSPAIPHGSRVKIRMDTPSGVKDSIPAWIKF
Maize 223 DFNNWNPNADAMARNEYGVWEIFLPNNADGSPAIPHGSRVKIRMDTPSGVKDSIPAWIKF
Sorghum 216 DFNNWNPNADAMTRNEYGVWEIFLPNNADGSPAIPHGSRVKIRMDTPSGVKDSIPAWIKF
Barley 214 DFNNWNPNADTMTRDDYGVWEIFLPNNADGSPAIPHGSRVKIRMDTPSGVKDSISAWIKF
Wheat 216 DFNNWNPNADTMTRDDYGVWEIFLPNNADGSPAIPHGSRVKIRMDTPSGVKDSISAWIKF
AmyAc_bac_euk_BE
sug-h 293 AVQAPGEIPYNGIYYDPPEEEKYVFQHPQPKRPNSLRIYESHIGMSSPEPKINTYANFRD
WT 293 AVQAPGEIPYNGIYYDPPEEEKYVFQHPQPKRPNSLRIYESHIGMSSPEPKINTYANFRD
Maize 283 SVQAPGEIPYNGIYYDPPEEEKYVFQHPQPKRPKSLRIYESHVGMSSPEPKINTYANFRD
Sorghum 276 SVQAPGEIPYNGIYYDPPEEEKYVFKHPQPKRPKSLRIYESHIGMSSPEPKINTYANFRD
Barley 274 SVQAPGEIPFNGIYYDPPEEEKYVFQHPQPKRPESLRIYESHIGMSSPEPKINSYANFRD
Wheat 276 SVQAPGEIPFNGIYYDPPEEEKYVFQHPQPKRPESLRIYESHIGMSSPEPKINSYANFRD
sug-h 353 EVLPRIKKLGYNAVQIMAIQEHSYYASFGYHVTNFFAPSSRFGTPEDLKSLIDKAHELGL
WT 353 EVLPRIKKLGYNAVQIMAIQEHSYYASFGYHVTNFFAPSSRFGTPEDLKSLIDKAHELGL
Maize 343 EVLPRIKKLGYNAVQIMAIQEHSYYASFGYHVTNFFAPSSRFGTPEDLKSLIDKAHELGL
Sorghum 336 EVLPRIKRLGYNAVQIMAIQEHSYYASFGYHVTNFFAPSSRFGTPEDLKSLIDKAHELGL
Barley 334 EVLPRIKRLGYNAVQIMAIQEHSYYASFGYHVTNFFAPSSRFGTPEDLKSLIDRAHELGL
Wheat 336 EVLPRIKRLGYNAVQIMAIQEHSYYASFGYHVTNFFAPSSRFGTPEDLKSLIDRAHELGL
sug-h 413 LVLMDIVHSHASNNTLDGLNGFDGTDTHYFHGGPRGHHWMWDSRLFNYGSWEVLRYLLSN
WT 413 LVLMDIVHSHASNNTLDGLNGFDGTDTHYFHGGPRGHHWMWDSRLFNYGSWEVLRYLLSN
Maize 403 LVLMDIVHSHSSNNTLDGLNGFDGTDTHYFHGGPRGHHWMWDSRLFNYGSWEVLRFLLSN
Sorghum 396 LVLMDIVHSHSSNNTLDGLNGFDGTDTHYFHGGPRGHHWMWDSRLFNYGSWEVLRYLLSN
Barley 394 LVLMDIVHSHSSNNTLDGLNGFDGTDTHYFHGGPRGHHWMWDSRLFNYGSWEVLRFLLSN
Wheat 396 LVLMDIVHSHSSNNTLDGLNGFDGTDTHYFHGGPRGHHWMWDSRLFNYGSWEVLRFLLSN
sug-h 473 ARWWLEEYKFDGFRFDGVTSMMYTHHDLQVAFTGNYGEYFGFATDVDAVVYLMLVNDLIH
WT 473 ARWWLEEYKFDGFRFDGVTSMMYTHHGLQVAFTGNYGEYFGFATDVDAVVYLMLVNDLIH
Maize 463 ARWWLEEYKFDGFRFDGVTSMMYTHHGLQVTFTGNYGEYFGFATDVDAVVYLMLVNDLIR
Sorghum 456 ARWWLEEYKFDGFRFDGVTSMMYTHHGLQVAFTGNYGEYFGFATDVDAVVYLMLVNDLIH
Barley 454 ARWWLEEYKFDGFRFDGVTSMMYTHHGLQMTFTGNYGEYFGFATDVDAVVYLMLVNDLIH
Wheat 456 ARWWLEEYKFDGFRFDGVTSMMYTHHGLQMTFTGNYGEYFGFATDVDAVVYLMLVNDLIH
*
sug-h 533 GLYPEAVAIGEDVSGMPTFCIPVQDGGVGFDYRLHMAVPDKWIELLKQSDEYWKMGDIVH
WT 533 GLYPEAVAIGEDVSGMPTFCIPVQDGGVGFDYRLHMAVPDKWIELLKQSDEYWKMGDIVH
Maize 523 GLYPEAVSIGEDVSGMPTFCIPVQDGGVGFDYRLHMAVPDKWIELLKQSDEYWEMGDIVH
Sorghum 516 GLYPEAVSIGEDVSGMPTFCIPVQDGGVGFDYRLHMAVPDKWIELLKQSDEYWKMGDIVH
Barley 514 GLYPDAVSIGEDVSGMPTFCIPVPDGGVGFDYRLHMAVADKWIELLKQSDESWKMGDIVH
Wheat 516 GLYPDAVSIGEDVSGMPTFCIPVPDGGVGFDYRLHMAVADKWIELLKQSDESWKMGDIVH
sug-h 593 TLTNRRWSEKCVTYAESHDQALVGDKTIAFWLMDKDMYDFMALDRPSTPRIDRGIALHKM
WT 593 TLTNRRWSEKCVTYAESHDQALVGDKTIAFWLMDKDMYDFMALDRPSTPRIDRGIALHKM
Maize 583 TLTNRRWLEKCVTYCESHDQALVGDKTIAFWLMDKDMYDFMALDRPSTPRIDRGIALHKM
Sorghum 576 TLTNRRWLEKCVTYCESHDQALVGDKTIAFWLMDKDMYDFMALDRPSTPVIDRGIALHKM
Barley 574 TLTNRRWLEKCVTYAESHDQALVGDKTIAFWLMDKDMYDFMALDRPSTPRIDRGIALHKM
Wheat 576 TLTNRRWLEKCVTYAESHDQALVGDKTIAFWLMDKDMYDFMALDRPSTPRIDRGIALHKM
sug-h 653 IRLVTMGLGGEGYLNFMGNEFGHPEWIDFPRGPQSLPNGSVLPGNNYSFDKCRRRFDLGD
WT 653 IRLVTMGLGGEGYLNFMGNEFGHPEWIDFPRGPQSLPNGSVLPGNNYSFDKCRRRFDLGD
Maize 643 IRLVTMGLGGEGYLNFMGNEFGHPEWIDFPRGPQSLPNGSVIPGNNNSFDKCRRRFDLGD
Sorghum 636 IRLITMGLGGEGYLNFMGNEFGHPEWIDFPRGPQSLPNGSVIPGNNYSFDKCRRRFDLGD
Barley 634 IRLVTMGLGGEGYLNFMGNEFGHPEWIDFPRGPQTLPTGKVLPGNNNSYDKCRRRFDLGD
Wheat 636 IRLVTMGLGGEGYLNFMGNEFGHPEWIDFPRGPQTLPTGKVLPGNNNSYDKCRRRFDLGD
Alpha-amylase_C
sug-h 713 ADYLRYHGMQEFDQAMQHLEEKYGFMTSEHQYISRKHEEDKVIIFERGDLVFVFNFHWSN
WT 713 ADYLRYHGMQEFDQAMQHLEEKYGFMTSEHQYISRKHEEDKVIIFERGDLVFVFNFHWSN
Maize 703 ADYLRYRGMQEFDQAMQHLEGKYEFMTSDHSYVSRKHEEDKVIIFERGDLVFVFNFHWSN
Sorghum 696 ADYLRYRGMQEFDQAMQHLEGKYEFMTSDHSYVSRKHEEDKVIIFERGDLVFVFNFHWSN
Barley 694 ADFLRYRGMQEFDQAMQHLEEKYGFMTSEHQYVSRKHEEDKVIIFERGDLVFVFNFHWSN
Wheat 696 ADFLRYRGMQEFDQAMQHLEEKYGFMTSEHQYVSRKHEEDKVIIFERGDLVFVFNFHWSN
sug-h 773 SYFDYRVGCLKPGKYKIVLDSDDGLFGGFSRLDHDAEYFTADWPHDNRPCSFSVYTPSRT
WT 773 SYFDYRVGCLKPGKYKIVLDSDDGLFGGFSRLDHDAEYFTADWPHDNRPCSFSVYTPSRT
Maize 763 SYFDYRVGCFKPGKYKIVLDSDDGLFGGFSRLDHDAEYFTADWPHDNRPCSFSVYAPSRT
Sorghum 756 SYFDYRVGCFKPGKYKIVLDSDDGLFGGFSRLDHDAEYFTADWPHDNRPCSFSVYAPSRT
Barley 754 SFFDYRVGCSKPGKYKVALDSDDALFGGFSRLDHDVDYFTTEHPHDNRPRSFSVYTPSRT
Wheat 756 SFFDYRVGCSRPGKYKVALDSDDALFGGFSRLDHDVDYFTTEHPHDNRERSFSVYTPSRT
sug-h 833 AVVYALTEDZ--
WT 833 AVVYALTEDZ--
Maize 823 AVVYAPAGAEDE
Sorghum 816 AVVYAPAGEEDE
Barley 814 AVVYALTE----
Wheat 816 AVVYALTE----
