# Supplementary material for: Sugary Endosperm is Modulated by Starch Branching Enzyme IIa in Rice (Oryza sativa L.)
Source: Rice (N Y). 2017 Jul 20;10:33. doi: 10.1186/s12284-017-0172-3 (PMC5519516; doi:10.1186/s12284-017-0172-3)
Supplement: Supplementary file 5 — Molecular markers used for fine mapping of the sug-h mutant. (DOCX 14 kb) [file 12284_2017_172_MOESM5_ESM.docx]

**Table S2**

| **Marker** | **Chr.** | **Type** | **Forward primer (5’-3’)** | **Reverse primer (5’-3’)** |
| --- | --- | --- | --- | --- |
| S08105 | 8 | STS | CCGTGCATATAGAGGAAAACG | ACACTCACACGTCATGAGCA |
| S08106 | 8 | STS | TTACGGATTGTCACGGTTTT | GGAATTTGTCACTGGTTTCCA |
| S08107 | 8 | STS | TTGGTAATGCCCATGCTAGA | CACGATTCGGTCATTTCAGA |
| S04056A | 4 | STS | CTGATTGCTCCCCTGAAGAG | TGCCTGCACTTGAAATCAGA |
| S04056B | 4 | STS | CCCTTGGATCGAATCGCTTC | CACGTCCTTCGGCGATTCAG |
| S04056C | 4 | STS | GTTGTTTCTCGTGAGGTGTTAT | AATTTCACACTGTGGTTGTTTA |
| AL731A | 4 | dCAPS/*Hha*I | GAATGCCCCTTAGGGTCAAAAGCTTTTG**C**G | AGATGAGATGCCCTGACCAAAT |
| AL731B | 4 | dCAPS/*Taq*I | ATAGCGTTGGTGTTTAGTACAGCTTA**T**C | CATGTGTCCTAGAAGAGTGCAA |
| S04057 | 4 | STS | GCGTCAGCGGCGCATTATCC | CAGTAGCTGACCGTCTCACG |
| S04058 | 4 | STS | GATCCATGCAGTTGATTGTGA | TCGTCTTATCTAAAAAGAAAATTTGA |
